# Supplementary material for: Modeling Adsorption of CO2 in Rutile Metallic Oxide Surfaces: Implications in CO2 Catalysis
Source: Molecules. 2023 Feb 13;28(4):1776. doi: 10.3390/molecules28041776 (PMC9961115; doi:10.3390/molecules28041776)
Supplement: Supplementary file 1 [file molecules-28-01776-s001.zip › molecules-2165080-supplementary.pdf]

Supporting Information

Modelling adsorption of CO_2 in rutile-metallic oxide surfaces; implications in CO_2 catalysis

Rogelio Chávez-Rocha [1], Itzel Mercado-Sánchez[1], Ismael Vargas-Rodriguez[1], Joseelyne Hernández-Lima[1], Adán Bazán-Jiménez [1], Juvencio Robles[2] and Marco A. García-Revilla [1,*]

[1] Chemistry Department, Natural and Exact Sciences Division, University of Guanajuato, Noria Alta S/N, Guanajuato 36050, Mexico

[2] Pharmacy Department, Natural and Exact Sciences Division, University of Guanajuato, Noria Alta S/N, Guanajuato 36050, Mexico

[*] Correspondence: magarcia@ugto.mx; Tel.: +52 473 732 00 06 (ext. 8120)

Conformation Models

For the construction of the surface finite models, the bulk structure of the metal oxide was used as the starting point, the crystallographic parameters were taken from the databank of BAND software. Then, the bulk structure was optimized using BAND software with the PBEsol/TZP level of theory. Finally, surfaces were obtained from Miller's indices (001) and (110), using the tool to build BAND slabs.

Adsorbates where individually optimized using B3LYP/TZP level of theory. Afterwards, a slab of optimized adsorbate was generated Then, this slab was used to perform the optimizations over the surface-slab from the optimized bulk solid. We placed both slabs together constraining only the metallic oxide coordinates, the initial distance between adsorbate molecules and surfaces was 5 Å. Different initial adsorbate orientations were tested, resulting similar optimized geometries, the conformation with lowest energy were used to perform the analysis.

In steps:

1. Obtaining crystallographic parameters of each oxide
2. Bulk structure optimization
3. Obtaining surfaces from drawings (001) and (110)
4. Creation of an adsorbate sheet from previously optimized molecular coordinates.
5. Conjunction of the system of both layers, varying the orientation of the adsorbate, but always at 5 Å between one of the oxygens and the most exposed metal atom.
6. The geometry of the entire system was optimized.
7. Similar conformations were obtained and those with lower energy were taken for analysis.

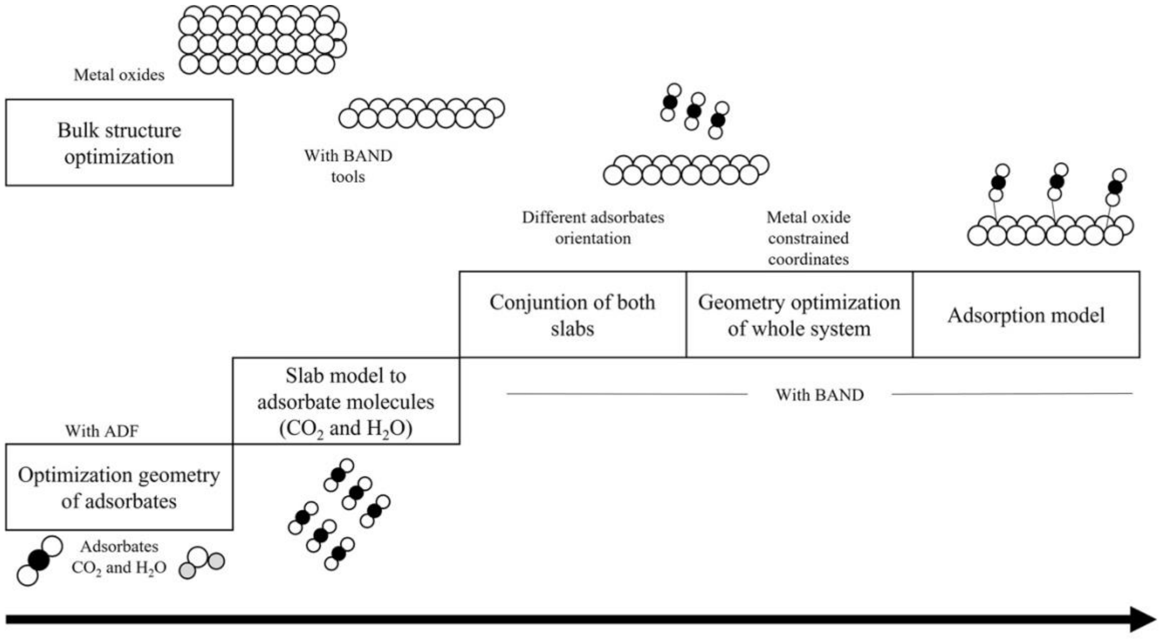

Figure S1. Scheme of models formation

Relative energies for CO_2 adsorbate

For the calculation of the relative energies of the adsorbed species of CO2, the coordinates were taken only of the adsorbed CO_2 molecule (without the surface) and a single point was made to calculate the energy of this species in isolation. Finally, the relative energy was calculated Δ using the following equation:

$$\Delta = E_{linear} - E_{adsorbed}$$

where E_{linear} is the optimized linear CO_2 energy and $E_{adsorbed}$ corresponds to the energy of the isolated CO_2 using the adsorbed geometry. Both using the same level of theory, B3LYP/TZP.

Coordinates of adsorption models (**xyz**)

GeO_2
001
9
:

Ge	0.000000	0.000000	0.000000
Ge	2.198750	2.198750	1.431250
O	3.561975	0.835525	1.431250
O	0.835525	3.561975	1.431250
O	1.363225	1.363225	0.000000
O	3.034275	3.034275	0.000000
O	1.988666	2.017206	-2.177299
O	0.406506	0.425539	-1.724123
C	1.338287	1.356105	-1.434277
VEC1	4.397500	0.000000	0.000000
VEC2	0.000000	4.397500	0.000000

110
9
:

Ge	0.000000	0.000000	0.000000
Ge	1.431250	3.109502	0.000000
O	1.431250	1.181611	0.000000
O	1.431250	-1.181611	0.000000
O	0.000000	3.109502	-1.181611
O	-0.018519	3.109502	1.196900
O	0.609601	2.195568	3.448966
O	-0.040678	0.542596	1.994611
C	0.228608	1.661918	2.475090
VEC1	2.862500	0.000000	0.000000
VEC2	0.000000	6.219004	0.000000

TiO_2
001
9
:

Ti	0.000000	0.000000	0.000000
Ti	-2.295000	-2.295000	-1.480000
C	0.727451	2.047956	1.039935
O	0.928012	3.241978	1.308355
O	-0.087345	1.234810	1.586085
O	-0.918000	0.918000	-1.480000
O	0.918000	-0.918000	-1.480000
O	1.377000	1.377000	0.000000
O	-1.377000	-1.377000	0.000000
VEC1	4.590000	0.000000	0.000000
VEC2	0.000000	4.590000	0.000000

110
9
:

Ti	0.000000	0.000000	0.000000
Ti	1.480000	3.245620	0.000000
O	1.480000	1.267957	0.000000
O	1.480000	-1.267957	0.000000
O	0.000000	3.245620	-1.267957
O	-0.129954	3.245620	1.379292
O	0.367897	2.380495	-3.411410
O	-0.011446	0.816426	-1.836590
C	0.130672	2.029676	-2.300759
VEC1	2.960000	0.000000	0.000000
VEC2	0.000000	6.491240	0.000000

SnO_2
001
9
:

Sn	0.000000	0.000000	0.000000
Sn	-2.416168	-2.416168	-1.621610
O	0.930648	-0.930648	-1.621610
O	-0.930648	0.930648	-1.621610
O	-1.485520	-1.485520	0.000000
O	1.485520	1.485520	0.000000
O	1.012342	3.342968	1.339674
O	-0.002548	1.345436	1.613970
C	0.825209	2.151772	1.037997
VEC1	4.832336	0.000000	0.000000
VEC2	0.000000	4.832336	0.000000

110
9
:

Sn	0.000000	0.000000	0.000000
Sn	1.621610	3.416978	0.000000
O	1.621610	5.517820	0.000000
O	1.621610	1.316135	0.000000
O	0.000000	3.416978	1.316135
O	0.000000	3.416978	-1.316135
O	0.727390	2.561550	3.427756
O	-0.081998	0.967575	2.018905
C	0.253987	2.114006	2.437645
VEC1	3.243221	0.000000	0.000000
VEC2	0.000000	6.833955	0.000000

IrO_2
001
9
:

Ir	0.000000	0.000000	0.000000
Ir	2.250000	2.250000	1.575000
O	3.600000	0.900000	1.575000
O	0.900000	3.600000	1.575000
O	1.350000	1.350000	0.000000
O	3.150000	3.150000	0.000000
O	1.927013	1.848926	-3.326936
O	0.354442	0.265928	-2.599437
C	1.149137	1.065794	-2.945619
VEC1	4.500000	0.000000	0.000000
VEC2	0.000000	4.500000	0.000000

110
9
:

Ir	0.000000	0.000000	0.000000
Ir	1.575000	3.181981	0.000000
O	1.575000	1.272792	0.000000
O	1.575000	-1.272792	0.000000
O	0.000000	3.181981	-1.272792

O	0.011667	3.181981	1.263364
O	1.208764	2.033574	4.584765
O	0.177496	0.328938	3.349394
C	0.692114	1.186711	3.963311
VEC1	3.150000	0.000000	0.000000
VEC2	0.000000	6.363961	0.000000

PbO_2
001
9
:

Pb	0.000000	0.000000	0.000000
Pb	-2.475000	-2.475000	-1.690000
O	-0.841500	0.841500	-1.690000
O	0.841500	-0.841500	-1.690000
O	1.633500	1.633500	0.000000
O	-1.633500	-1.633500	0.000000
O	0.930767	1.117927	5.030431
O	-0.541206	-0.399656	4.016695
C	0.194994	0.359508	4.524144
VEC1	4.950000	0.000000	0.000000
VEC2	0.000000	4.950000	0.000000

110
9
:

Pb	0.000000	0.000000	0.000000
Pb	1.690000	3.500179	0.000000
O	1.690000	1.190061	0.000000
O	1.690000	-1.190061	0.000000
O	0.000000	3.500179	-1.190061
O	0.000000	3.500179	1.190061
O	1.015361	2.571208	3.768224
O	-0.069238	0.811198	2.663286
C	0.465920	1.734147	3.171294
VEC1	3.380000	0.000000	0.000000
VEC2	0.000000	7.000357	0.000000

TiO2 (anatase)

001
9
:

Ti	3.784200	2.838150	1.189325
Ti	1.892100	2.838150	3.567975
O	1.892100	2.838150	1.587987
O	1.892100	4.730250	3.966637
O	3.784200	2.838150	3.169313
O	3.784200	0.946050	0.790663
O	4.624152	4.182623	-2.841981
O	3.369320	2.826628	-1.401349

C	3.999828	3.521925	-2.107576
VEC1	3.784200	0.000000	0.000000
VEC2	0.000000	3.784200	0.000000

101
9
:

Ti	-1.661377	-1.164828	-0.644461
Ti	1.914174	3.011940	-2.305669
O	-1.661377	-3.186791	-3.688248
O	-1.661377	2.311386	-2.027041
O	1.914174	2.391084	-5.906712
O	1.914174	-0.464274	-0.923089
O	1.024931	-0.030518	6.719740
O	-1.472794	-1.963259	3.586552
C	-0.310528	-0.904740	5.188929
VEC1	3.784200	0.000000	0.000000
VEC	2.000000	1.892100	5.119760

H2O

GeO_2
001
9
:

Ge	0.000000	0.000000	0.000000
Ge	2.198750	2.198750	1.431250
O	3.561975	0.835525	1.431250
O	0.835525	3.561975	1.431250
O	1.363225	1.363225	0.000000
O	3.034275	3.034275	0.000000
O	0.072339	0.052012	-1.778953
H	1.729982	1.765203	-0.821260
H	-0.596355	-0.606728	-2.064737
VEC1	4.397500	0.000000	0.000000
VEC2	0.000000	4.397500	0.000000

110
9
:

Ge	0.000000	0.000000	0.000000
Ge	1.431250	3.109502	0.000000
O	1.431250	1.181611	0.000000
O	1.431250	-1.181611	0.000000
O	0.000000	3.109502	-1.181611
O	-0.018519	3.109502	1.196900
O	-0.052618	0.300590	-1.863050
H	-0.005357	2.235366	-1.709441
H	0.847965	0.082987	-2.212688
VEC1	2.862500	0.000000	0.000000
VEC2	0.000000	6.219004	0.000000

TiO_2

001
9
:

Ti	0.000000	0.000000	0.000000
Ti	2.295000	2.295000	1.480000
O	3.693419	0.896581	1.480000
O	0.896581	3.693419	1.480000
O	1.398419	1.398419	0.000000
O	3.191581	3.191581	0.000000
O	1.268441	-0.720110	-1.538248
H	1.656693	0.151499	-1.780781
H	2.060788	-1.113081	-0.906139
VEC1	4.590000	0.000000	0.000000
VEC2	0.000000	4.590000	0.000000

110
9
:

Ti	0.000000	0.000000	0.000000
Ti	1.480000	3.245620	0.000000
O	1.480000	1.267957	0.000000
O	1.480000	-1.267957	0.000000
O	0.000000	3.245620	-1.267957
O	-0.129954	3.245620	1.379292
O	-0.025547	0.121554	-1.853370
H	-0.019950	2.468815	-1.876539
H	0.875696	-0.108819	-2.193519
VEC1	2.960000	0.000000	0.000000
VEC2	0.000000	6.491240	0.000000

SnO_2

001
9
:

Sn	0.000000	0.000000	0.000000
Sn	-2.416168	-2.416168	-1.621610
O	0.930648	-0.930648	-1.621610
O	-0.930648	0.930648	-1.621610
O	-1.485520	-1.485520	0.000000
O	1.485520	1.485520	0.000000
O	-0.095423	-0.093921	1.975813
H	0.581889	0.540974	2.293133
H	-1.833562	2.957913	0.834281
VEC1	4.832336	0.000000	0.000000
VEC2	0.000000	4.832336	0.000000

110

9
:

Sn	0.000000	0.000000	0.000000
Sn	1.621610	3.416978	0.000000
O	1.621610	5.517820	0.000000
O	1.621610	1.316135	0.000000
O	0.000000	3.416978	1.316135
O	0.000000	3.416978	-1.316135
O	3.308255	0.454880	1.994497
H	2.427385	0.243192	2.380686
H	3.249359	2.535486	1.803379
VEC1	3.243221	0.000000	0.000000
VEC2	0.000000	6.833955	0.000000
